# Editorial Notes and Miscellany

**Published:** 1912-02

**Authors:** 


### Editorial Notes and Miscellany.

ALL that blisters is not bliss.

---

KEEP her coming. She gets better every issue.

C. V. DOWNS, M. D.

Colmar, New Mexico.

---

DR. WILEY was unanimously acquitted by the congressional committee of those absurd charges of "conspiracy" brought by his enemies.

---

DR. ISAAC SMITH of Rogers, Texas, died of meningitis at Rogers, January 15 (ult.). It is said that he contracted the disease from patients he had been attending.

---

THE TOTAL number of medical colleges in the United States and Canada is 450; the total number of hospitals and sanitariums, 5400; the total number of medical journals, 225, and the total number of public medical libraries, 100.

---

PLEASE keep it coming. No journal comes to my desk that is enjoyed more than the "Red Back." With best wishes for a prosperous New Year.

L. H. REEVES.

Decatur, Texas.

---

A HARVEST FOR THE DRUGGISTS.—The boards of health having advised spraying nose and fauces with a germicide, as a preventive of meningitis, the demand for atomizers has been something un-

*The *diplococcus intercellularis meningitidis* was discovered in 1887 by Weichselbaum, and proved by Jager in 1895 to be the cause of cerebro-spinal meningitis, and found, during the disease, in the cerebro-spinal fluid.

precedented and has created a famine in that article. One drug firm in Waco is said to have sold 150 dozen in one day!

---

| | |
|---|---|
| TEXAS MEDICAL JOURNAL one year | $1 00 |
| *American Journal Clinical Medicine* | 2 00 |
| "Strange Case of Dr. Bruno" | 1 50 |
| | $4 50 |

All for $3.00. Renewals count if arrears are paid.

---

THE BED BUG'S OPPORTUNITY.—The mad dog harbors rabies; the felines, too, they say. The pesky rat,—for all of that, is lined up in the fray,—as host of plague-bubonic! Oh, were I but Byronic I'd speak of ties platonic twixt men and pathogenics of today! "Now by the rood," as Hamlet says, it grieves me sore to say, that *Cimex* has been slighted; has never been indicted in any kind of way as cause of any "itis," yet we know well that his bite is—of a kind to cause dismay! *Pediculosus corporis* of typhoid is accused, Oh, *Cimex,* friend, butt in, and when famous you have grown, you can tell those other fellows you've adopted,—the *meningo-coccus* for your own.

P. M. P.

Austin, Texas.

---

THE NEW SURGEON GENERAL of the United States Public Health and Marine Hospital Service is Dr. Rupert Blue. He is a native of South Carolina, born in 1868, and graduated from the University of Maryland in 1892. He first entered the Marine Hospital Service as an interne in 1892 and was commissioned assistant surgeon in 1893. He was promoted to surgeon May 1, 1909. He served under M. H. S. Surgeon White at New Orleans in 1905, in the yellow fever outbreak. In 1903-1904 he also served with Dr. White in San Francisco, combating the bubonic plague. In 1907 he was director of sanitation at the Jamestown Exposition. In all these positions he gave evidence of executive ability of a high order. He graduated later from the London School of Tropical Medicine. He is said to be a young man of pleasing personality and is deemed a fit successor to the lamented Walter Wyman.

---

KETTLE AND POT.—The irascible and vengeful editor of the *Journal American Medical Association,* the notorious Simmons, makes a fierce attack editorially on the *American Journal of Surgery* in the December 16, 1911, issue of the Octopus (*J. A. M. A.*), covering three columns; and over the shoulders of the editor of the *American Journal of Surgery* lashes the whole independent medical press for carrying advertisements of proprietaries not censored by ye immaculate Council of Pharmacy. Dr. Brickner, editor of the *Journal of Surgery,* in his January number, shows that his critic is carrying advertisements of the same class—ads that are open to the same criticisms he bestows on others, and intimates that ye editor of the Octopus is actuated by personal spite or prejudice. It is a case of mote and beam, or kettle and pot. Dr. Simmons might as well whistle to the wind as to insist that physicians shall not prescribe the better class of proprietary medicines, or if they *do,* they must call Listerine, for example, *Liquor Antisepticus, Antiphlogistine, Emp. Kaolin Comp.* Rot!

